# The Impact of Ketamine on Outcomes in Acute Pain Management: An Umbrella Review

**DOI:** 10.3390/jcm13247699

**Published:** 2024-12-17

**Authors:** Dmitriy Viderman, Diyara Mukazhan, Kamilla Kapessova, Meiram Tungushpayev, Rafael Badenes

**Affiliations:** 1 Department of Surgery, School of Medicine, Nazarbeyv University, Kerei and Zhanibek Khandar Str. 5/1, Astana 010000, Kazakhstan; diyara.mukazhan@alumni.nu.edu.kz (D.M.); kamilla.adileva@nu.edu.kz (K.K.); meiram.tungushpayev@alumni.nu.edu.kz (M.T.); 2 Departement of Anesthesiology, Intensive Care, and Pain Medicine, National Research Oncology Center, Kerei and Zhanibek Khandar Str. 3, Astana 010000, Kazakhstan; 3 Department of Anaesthesiology and Intensive Care, Hospital Clìnico Universitario de Valencia, University of Valencia, 46003 Valencia, Spain; rafaelbadenes@gmail.com

**Keywords:** ketamine, acute pain, analgesia, pain management, pain medicine, N-methyl-D-aspartate receptor, outcomes

## Abstract

**Background/Objectives**: Ketamine offers effective pain relief with fewer side effects than traditional analgesics, making it a promising alternative for acute pain treatment. However, further research is needed to fully assess its role in perioperative care. This umbrella review aimed to compile the highest-quality evidence available regarding the application of ketamine in managing acute pain. **Methods**: A thorough search of the literature was carried out in PubMed, Scopus, and the Cochrane Library, including systematic reviews that focused on the application of ketamine in managing acute pain. The data extraction included the research type, analgesics used, number of studies and patients per review, pain types, scoring methods, ketamine doses, administration routes, and reporting guidelines. **Results**: Of the 807 records identified, 20 studies met the inclusion criteria. In accordance with the AMSTAR-2 evaluation, most of the systematic reviews were rated as critically low quality. Intravenous ketamine administered during the perioperative period was found to reduce the pain intensity of acute pain within 15–30 and 60 min following treatment, and decrease postoperative opioid consumption by 14–50% at both 24 and 48 h after surgery. **Conclusions**: Evidence shows that intravenous ketamine reduces the pain intensity, postoperative opioid use, and the risk of vomiting and nausea while improving analgesia, making it a valuable adjunct in perioperative pain management.

## 1. Introduction

Acute postoperative pain is a prevalent and significant clinical issue, affecting a substantial proportion of patients undergoing surgical procedures. Reports indicate [1] that approximately one in five patients experience severe pain during the day following the surgery.

Effective pain management is crucial for optimal perioperative outcomes, making the prevention and management of postoperative pain one of the key focus for perioperative specialists. Prolonged and intense acute pain can lead to a series of adverse effects, such as sleep disturbances and impaired physical functioning, further hindering postoperative recovery and rehabilitation [2]. Additionally, it can adversely affect psychological functioning, causing increased stress responses, anxiety, and catastrophizing, which, in turn, may elevate the risks of developing chronic or persistent postsurgical pain (PPSP) [3]. Although preemptive analgesia seeks to minimize the likelihood of acute pain resulting to chronic pain, traditional analgesics, such as paracetamol, alone may not be adequate for managing pain in the acute postoperative phase [4].

Despite advances in analgesic techniques, acute pain management is fraught with challenges. Numerous barriers impede effective pain control, including fear and negative attitudes toward the use of opioids due to insufficient physician training and patient education, along with multiple side effects related to current pain relief therapies [5]. Traditional pain treatment, such as opioid medications, nonsteroidal anti-inflammatory drugs (NSAIDs), and regional anesthetic interventions, might not be suitable due to contraindications, limited efficacy, and undesirable side effects [1]. Although opioids significantly reduce the pain intensity, they may result in a range of side effects, including nausea, vomiting, respiratory depression, ileus or constipation, and urinary retention [6]. While NSAIDs have the potential to decrease morphine usage in intravenous patient-controlled analgesia (IV PCA), they may also lead to anastomotic leaks, gastrointestinal bleeding, renal impairment, and cardiovascular events [7,8]. These adverse effects can result in the discontinuation of therapy and hinder dosing at maximum efficacy. Additionally, effective alternatives to opioids would be advantageous for specific patient groups, including the elderly, individuals without prior opioid exposure, long-term opioid users, individuals with a history of opioid abuse, and former opioid- and alcohol-dependent individuals [9].

Given these limitations, there is a critical imperative for pain control agents that can provide substantial pain relief with a more favorable side effect profile. A multimodal analgesic approach targeting multiple anatomical pain pathways has been widely considered as a new approach to replace traditional analgesics and showed promising results in clinical practice [10]. One of the most widely known anesthetics that offers this multimodal approach is ketamine, a non-competitive “N-methyl-D-aspartate (NMDA) receptor antagonist” that can potentially reduce the reliance on opioids [11]. Moreover, ketamine was shown to have a rapid pharmacological response and the ability to provide sustained pain relief with minimal respiratory depression, making it a valuable adjuvant in the perioperative setting [12].

In addition to perioperative pain management, ketamine is also studied for its efficacy in managing acute pain in emergency departments. Acute pain in the emergency medicine unit is a common and challenging issue, with patients presenting with various painful conditions that require prompt and effective pain relief [13]. Ketamine, combined with conventional analgesics, was found to provide rapid analgesia with a good safety profile in this setting [14]. Moreover, it demonstrated superior analgesic effects compared with opioids for both chronic and acute pain [9]. This makes ketamine a valuable option for acute pain management in emergency care facilities, where fast-acting and effective pain relief is crucial.

The high prevalence of acute postoperative pain and its negative impact on perioperative outcomes underscore the need for improved pain management strategies. Although prior reviews highlighted ketamine as a promising complementary drug for managing acute postoperative pain, its use is not without risks. The drug is associated with notable adverse events, which, like its analgesic effects, are dose-dependent, and the most effective dosage and administration route have yet to be determined [4]. Ketamine’s potential to enhance analgesia while mitigating the adverse effects associated with traditional pain medications positions it as a promising alternative in the management of acute pain. Further research is needed to fully explore the benefits of ketamine in the management of acute pain, which is why this study aimed to explore and justify the role of ketamine in this important aspect of perioperative care. The objective of this umbrella review is to synthesize the highest available quality level of evidence related to the application of ketamine in the management of acute pain.

## 2. Materials and Methods

We followed “PRISMA (Preferred Reporting Items for Systematic Reviews and Meta-Analyses)” guidelines in conducting this umbrella review, supplemented by guidance on systematic reviews of systematic reviews. This process followed the protocol previously registered in the OSF database (https://osf.io/hmjwz (accessed on 15 November 2024), registration DOI: https://doi.org/10.17605/OSF.IO/HMJWZ).

The inclusion criteria were systematic reviews that studied ketamine for acute pain management. We did not limit our inclusion criteria to any specific types of acute pain, taking into consideration that many types of acute pain overlap with each other. There were no limitations in regard to the patient population presenting with acute pain, diseases and comorbidities, surgeries, procedures, and comparators. The exclusion criteria included study designs and publication types other than systematic reviews, systematic reviews unrelated to ketamine in pain management, or addressing conditions other than acute pain.

### Search Strategy

We used systematic searches in PubMed, Scopus, and Cochrane databases. The following search terms and their combinations were used: “esketamine” OR “ketamine” OR “ketamin” OR “ketamine s” OR “ketamines” AND “acute pain” OR (“acute” AND “pain” OR “acute pain”).

Data extraction covered various aspects, such research type, analgesic agents used, number of studies included in the SR, number of patients included in each SR, types of acute pain, pain-scoring method, doses of ketamine, route of administration, and reporting guidelines.

Due to including some overlapping studies and differences in reporting styles, we did not conduct a meta-analysis.

We used the AMSTAR-2 to assess the methodological quality of systematic reviews and meta-analyses [15].

## 3. Results

### 3.1. Characteristics of Included Systematic Reviews

**Figure 1:**
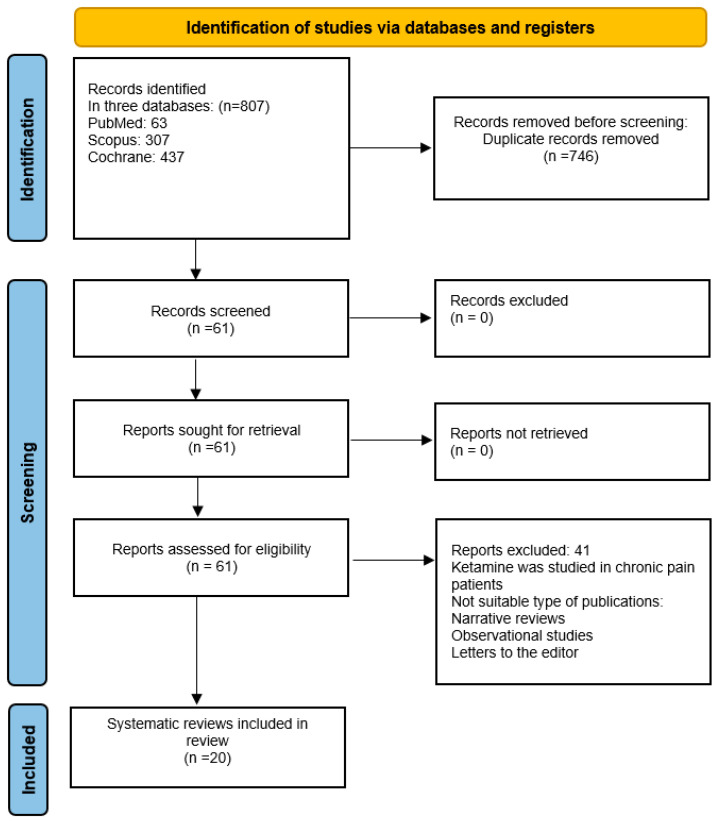
PRISMA diagram.

In total, 807 records were identified and 746 duplicates were removed. Sixty-one records were screened and 41 records were excluded after screening. Overall, 20 systematic reviews and meta-analyses were included in this umbrella review [4,16,17,18,19,20,21,22,23,24,25,26,27,28,29,30,31,32,33,34] (Figure 1), the basic characteristics of which are demonstrated in Table 1. Among these studies, 19 were systematic reviews of randomized controlled trials (RCTs) with meta-analyses [4,16,17,18,19,20,21,22,23,24,25,26,27,28,29,30,32,33,34], while one study by Xu et al. (2016) [31] was a systematic review without a meta-analysis. The selected systematic reviews (SRs) were published over an 18-year period from 2005 to 2023.

The number of studies reviewed within these SRs varied significantly. The study by Li and Chen (2019) [23] reviewed the fewest papers, with a total of six trials, and included the smallest patient population of 244 people. However, Yan and McLeod (2015) [33], despite having the same number of studies reviewed, had a larger total patient population of 932. On the other end of the spectrum, the study by Brink et al. (2018) [4] reviewed the highest number of studies, totaling 130, and included a total patient population of 8341.

According to the AMSTAR-2 assessment, the majority of SRs [16,17,18,19,20,21,22,23,25,26,27,28,29,30,31,32,33,34] were found to have critically low quality, with more than one critical flaw in the methodology (Table 2). Only one SR [4] was high quality according to the checklist, and had only one non-critical flaw.

Most reviews focused on comparing the effectiveness of ketamine against a control group, which was either a placebo or other analgesics, with Zhao et al. (2018) [34] being an exception by using a baseline pain score as the control. Additionally, some reviews did not focus solely on ketamine: Wertli et al. (2014) [30] and Xu et al. (2016) [31] investigated the effectiveness of various IV and/or oral analgesics, and Thompson et al. (2019) [27] reviewed different NMDA receptor antagonists. However, for this umbrella review, only information about ketamine was used.

In terms of pain-scoring methods, seven reviews utilized the Visual Analog Scale (VAS) [4,22,23,24,29,31,32], five used the Numeric Pain Rating Scale (NPRS) [16,25,26,27,29], and three employed both the VAS and NPRS [28,30,34]. Two reviews used both the Verbal Rating Scale (VRS) and VAS [17,18]; one used the Douleur Neuropathique 4 (DN4), along with the VAS [20]; and two reviews did not measure pain intensity at all [21,33].

Regarding the reporting guidelines, the majority of the studies that specified their guidelines followed “Preferred Reporting Items for Systematic Review and Meta-Analyses—PRISMA” (10 reviews). Additionally, one review used the QUOROM guidelines [17], and another used the “Methodological Expectations of Cochrane Intervention Reviews (MECIR) guidelines” [4].

### 3.2. Patient Population and Types of Pain

This umbrella review encompassed a diverse patient population with varying backgrounds, countries, and ages [4,16,17,18,19,20,21,22,23,24,25,26,27,28,29,30,31,32,33,34]. This review also addressed patients experiencing different types of acute pain, such as pain from various procedures in the emergency department and perioperative pain from undergoing surgical procedures, such as a total knee arthroscopy and knee replacement, spine surgery, and cesarean section [4,16,17,18,19,20,21,22,23,24,25,26,27,28,29,30,31,32,33,34].

### 3.3. Dose, Duration, Regiment, and Efficacy of Ketamine Therapy

Balzer et al. (2021) [16], in a study of low-dose ketamine (LDK) administered intravenously as a bolus or infusion for less than 30 min, revealed no significant difference between the effectiveness of LDK in concentrations of 0.2–0.5 mg/kg against 0.1 mg/kg morphine within the first 15 and 45–60 min after analgesia (MD = 0.52, 95% CI = −0.03 to 1.07), and a slight difference favoring morphine at 60–90 and 90–120 min demonstrated by levels of reported mean pain scores in the emergency department settings. Additionally, no significant difference was found in patients’ needs for rescue analgesics between these two groups. A pain assessment was performed using the NPRS at intervals from a quarter of an hour to two hours.

Bell et al. (2005) [17] reported that the use of ketamine in the perioperative settings reduced patient-controlled morphine consumption during the initial 24 h postoperative period, with a decrease in the weight mean difference of −15.98 mg. Furthermore, meta-analyses revealed the reduction in the need for the rescue analgesics by 30–50%. The same results were reported in the review by Bell et al. (2006) [18]. These two studies also aimed to determine the preferred dosage of ketamine and divided the doses from trials into four groups: 10 mg, 30 mg, 65 mg, and 250–270 mg per 70 kg over 24 h. It was also estimated that there was no significant decrease in morphine consumption with an increase in ketamine concentrations above 30 mg. Pain was measured with the VAS and the VRS over one year.

Brinck et al. (2018) [4] administered a 0.25–1.0 mg/kg bolus dose of racemic ketamine and 2–5 μg/kg/min via infusion and reported that the treatment resulted in a 19% reduction in postoperative opioid consumption at both 24 and 48 h after surgery. Pain scores assessed by the VAS showed a 19% decrease at rest after 24 h and a 14% decrease after 48 h compared with the control group treated with a placebo. Additionally, pain scores during movement decreased by 22% after 24 h and by 16% after 48 h. Notably, ketamine was more effective for patients with moderate and severe acute pain. Moreover, the time to the first analgesic request after surgery increased by 22 min in the ketamine-treated group.

Galili et al. (2023) [19] suggested that adjuvant single-dose ketamine (SDK) administration reduced mean pain intensity scores by more than one NPRS point within 15–30 and 60 min after the treatment of acute pain in emergency department settings compared with the use of opioids alone. Furthermore, treatment with adjuvant SDK had a higher patient satisfaction rate (8.57 ± 2.1) compared with the control group (6.05 ± 2.6), with a statistically significant difference (*p* = 0.01). The administration of adjuvant SDK also resulted in the lowered need in rescue analgesia after 10–20 min and 30–40 min (RR 0.40; 95% CI 0.21 to 0.75) after the treatment. The doses and routes of ketamine administration reviewed in this study were 0.1–0.3 mg/kg intravenously, 1.0 mg/kg by intranasal routes, and 1.5 mg/kg by inhalation.

Hannon et al. (2023) [20] presented inconsistent results in the pain relief efficacy of ketamine compared with a placebo within 48 h postoperatively. Thus, only a meta-analysis on the occurrence complications was performed. Postoperative pain ratings were determined using the VAS and the DN4 questionnaire.

Li et al.’s (2021) [22] results demonstrated no statistically significant difference in mean pain score between ketamine (1 mg/kg) and IV opioids (1 mg/kg morphine or 1 mcg/kg fentanyl) 15 and 60 min after administration and better pain reduction with opioids at 30 min (MD: 1.09; 95% CI: 0.06, 2.13; I^2^ = 83%; *p* = 0.04) in emergency settings, with pain scores assessed using the VAS. Furthermore, a non-significant difference with a tendency for better pain relief was revealed when ketamine (25 mg fixed dose and 1 mg/kg) was compared with a placebo at 15 (MD: −0.90; 95% CI: −2.34, 0.54; I^2^ = 94%; *p* = 0.22) and 60 min (MD: −1.47; 95% CI: −3.04, 0.10; I^2^ = 71%; *p* = 0.07). However, at 30 min, the ketamine efficacy had a high statistical significance with MD: −0.82; 95% CI: −1.43, −0.20; I^2^ = 64%; *p* = 0.009. Demand for the rescue analgesics in the ketamine group was increased in the study with opioids (OR: 4.69; 95% CI: 1.75, 12.60; *p* = 0.02) and decreased in the study with a placebo (OR: 0.36; 95% CI: 0.16, 0.80; I^2^ = 66%; *p* = 0.01) as a control.

Li and Chen (2019) [23] reported that the VAS pain scores in the ketamine group were significantly lower than in the control group treated with a placebo at 6 h (WMD: −0.296, 95% CI: −0.488 to −0.104, *p* = 0.003), 12 h (WMD: −0.304, 95% CI: −0.491 to −0.117, *p* = 0.001), and 24 h (WMD: −0.252, 95% CI: −0.404 to −0.101, *p* = 0.001) after a TKA, and no significant difference after 48 h (WMD: −0.007, 95% CI: −0.131 to 0.116, *p* = 0.911). Moreover, a significantly lower level of cumulative morphine consumption was noted at 24 (WMD: −17.402, 95% CI: −34.006 to −0.798, *p* = 0.040) and 48 h (WMD: −19.963, 95% CI: −34.056 to −5.871, *p* = 0.005) postoperatively in the ketamine group. Additionally, it was revealed that the type of analgesics prescribed had no significant effect on the duration of hospitalization (WMD: 0.070, 95% CI: −0.314 to 0.453, *p* = 0.722).

In the review performed by Meyer-Frießem et al. (2022) [24], it was estimated that IV ketamine administered via a bolus and/or infusion led to the lowered pain scores during movement at the early postoperative period (MD: −1.04, 95% CI: −1.51 to −0.57), as assessed using the VAS. Furthermore, patients in the ketamine group had lower mean oral opioid consumption by 97.3 mg after 24 h (95% CI: −164.8 to −29.7) and 186.4 mg after 48 h after surgery (95% CI: −347.6 to −25.2). Analyses also suggested a very low quality of evidence with risks of bias and inconsistency showing that ketamine administered with the initial bolus and during the surgery slightly reduced the pain intensity at rest (MD: −0.6 points, 95% CI: −2.01 to 0.8) and during movement (MD: −0.79 points, 95% CI: −1.22 to −0.36) at 24 h after surgery.

Pan et al. (2019) [25] included studies that implemented 0.5–1.0 mg/kg intra-articular and 0.01–0.15 mg/kg ketamine intravenously for patients undergoing total knee arthroscopy and compared its effectiveness with a control group of patients treated with a placebo. Analysis of these studies revealed a significant reduction in pain scores measured by the NPRS within 2 h after the surgery in the ketamine group (MD= −2.95, 95% CI= −3.36 to –2.54, I^2^ = 0%, *p* < 0.00001). Additional findings were the reduction in the consumption of analgesics (Std. MD= −1.03, 95% CI= −1.70 to –0.36, *p* = 0.002) and increased time before the first analgesic request (Std. MD = 1.21, 95% CI = 0.45–1.96, *p* = 0.002). It was also estimated by Pendi et al. (2018) [26] that the morphine-equivalent consumption levels were lower in a group treated with IV ketamine after 4 h, 8 h, and 12 h, with the most prominent drop at 24 h (MD: −14.38, 95% CI: −18.13 to −10.62, *p* < 0.001) after the spine surgery. However, this tendency was no longer apparent at 36 h (MD: −8.64, 95% CI: −18.62 to 1.33, *p* = 0.09), while at 48 h, this tendency was reversed, as the opioid consumption insignificantly increased in the ketamine group (MD: 2.39, 95% CI: −10.42 to 15.21, *p* = 0.71). Moving on to the reported pain scores measured with the NPRS, they were almost at the same level as the control immediately after the surgery (MD: −0.18, 95% CI: −0.69 to 0.33, *p* = 0.48), at 1 h (MD: −1.02, 95% CI: −2.46 to 0.42, *p* = 0.16), 24 h (MD: −1.27, 95% CI: −1.70 to −0.84, *p* < 0.001), at 36 h (MD: 0.15, 95% CI: −1.15 to 1.44, *p* = 0.83), 48 h (MD: −0.35, 95% CI: −0.96 to 0.26, *p* = 0.26), and 72 h (MD: 0.04, 95% CI: −0.36 to 0.43, *p* = 0.85) following the surgery. Nevertheless, at 6 (MD: −1.18, 95% CI: −1.67 to −0.69, *p* < 0.001) and 12 h (D: −1.01, 95% CI: −1.51 to −0.52, *p* < 0.001), compelling evidence of a lower mean pain score was demonstrated.

Thompson et al. (2019) [27] tested the effectiveness of different NMDAR antagonists to treat noxious stimuli-induced pain and included agents such as dextromethorphan, L-4 chlorokynurenine, neuramexane, CHF3381, and magnesium sulphate, along with ketamine, administered intravenously, intramuscularly, orally, subcutaneously, or as a topical gel in a mean concentration of 0.34 mg/kg (range 0.03–1.00). The efficacies of the analgesics were demonstrated through moderately decreased pain intensity with both racemic (SMD = 0.57; 95% CI: 0.40, 0.74; *p* < 0.001) and S (+) (SMD = 0.69; 95% CI: 0.35, 1.03; *p* = 0.001) IV ketamine, mildly increased pain tolerance (k = 9; SMD 0.46; 95% CI 0.19, 0.72; *p* = 0.004), slightly increased pain tolerance (k= 30; SMD 0.31; 95% CI 0.17, 0.45; *p*< 0.001), and a gentle increase in secondary hyperalgesic area size compared with the control (k = 15; SMD 0.54; 95% CI 0.34, 0.74; *p* < 0.001), as assessed by the NPRS.

Wang et al. (2020) [28], in their review, analyzed the overall ketamine effectiveness and reported significance regarding pain relief and its effectiveness to reduce pain scores in two types of analgesia, general and spinal, against a control group. Also, the analysis revealed a more significant reduction in pain scores measured by the VAS than by the NRS. The pain scores were evaluated from 2 h to 6 weeks postoperatively. The ketamine group had a noticeable reduction in pain relief drugs; the same trend was observed in morphine consumption rates during spinal anesthesia, but not in the group of patients under general anesthesia. According to this study, the decreases in the mean consumption rates were 6.11 mg for morphine (95% CI, 2.29–9.93; *p* =0.002) and 35.46 mg for other pain-relieving agents (95% CI, 21.61–49.31; *p* < 0.00001). Additionally, the analysis demonstrated a significantly prolonged time for the first analgesics request in the ketamine group (MD, 72.48; 95% CI, 50.85–94.11; *p* < 0.00001) and spinal anesthesia (MD, 70.50; 95% CI, 49.20, 91.79; *p* < 0.00001), but insufficient in the general anesthesia group (MD, 232.73; 95% CI, –54.61, 520.08; *p* = 0.11).

As reported by Wang et al. (2020) [29], the VAS scores had a statistically significant drop in the ketamine group compared with a placebo at 6 h (WMD = −1.45, 95% CI: −1.71 to −1.18, *p* < 0.00001), 12 h (WMD = −1.55, 95% CI: −2.28 to −0.82, *p* < 0.0001), 24 h (WMD = −0.78, 95% CI: −1.25 to −0.31, *p* = 0.001), and 48 h after a TKA or THA (WMD = −0.74, 95% CI: −1.26 to −0.22, *p* = 0.006). A meta-analysis of pooled results demonstrated a lowered amount of morphine equivalent consumption at 24 h (WMD = −17.58, 95% CI: −29.07 to −6.10, *p* = 0.003) and 48 h after the surgery (WMD = −16.82, 95% CI: −27.75 to −5.89, *p* = 0.003).

The systematic review performed by Xu et al. (2016) [31] investigated the effectiveness of different IV therapies against complex regional pain syndrome (CRPS). Among these, ketamine resulted in complete pain relief in severe refractory CRPS when an intravenous anesthetic dose was used but demonstrated a low effectiveness when used in subanesthetic doses. The VAS was used to measure pain, with assessments spanning from 10 to 50 days.

Xu et al. (2019) [32] investigated the effectiveness of ketamine with different routes of administration and compared them to a placebo. A significant decrease in pain scores, rated by the VAS, was observed in patients treated with IV ketamine during the early postoperative period (WMD −1.21, 95% CI −1.45 to −0.98, *p* < 0.001), but not during the late postoperative period (WMD −0.48, 95% CI −1.13 to 0.17, *p* = 0.14). Meanwhile, a reversed trend was observed in the group with intra-articular ketamine administration with no changes in the pain score during early postoperative period (WMD −0.12, 95% CI −0.51 to 0.26, *p* = 0.52) and with a significant decrease during the late postoperative period (WMD −0.49, 95% CI −0.70 to −0.29, *p* < 0.001). Additionally, epidural ketamine (10 mg) resulted in a noticeable drop in pain intensity during the late postoperative time (WMD −2.10, 95% CI −3.30 to −0.90, *p* < 0.001). While intravenous ketamine demonstrated lowered the cumulative morphine consumption rates at both 24 h (WMD −17.76, 95% CI −31.25 to −4.27, *p* = 0.01) and 48 h (WMD −21.79, 95% CI −25.46 to −18.11, *p* < 0.001) after the surgery, intra-articular ketamine had a drop only at 48 h (WMD −4.10, 95% CI −5.85 to −2.35, *p* < 0.001), but not at 24 h postoperatively (WMD −0.40, 95% CI −1.83 to 1.03, *p* = 0.58).

Zhao et al. (2018) [34] presented results stating that the treatment of CRPS with ketamine leads to a decrease in mean pain scores compared with the baseline (*p* < 0.000001). Pain relief rates were also analyzed and were 69% (95% CI 53%, 84%) immediately and 58% (95% CI 41%, 75%) during a 1–3-month follow-up, as evaluated by the NPRS and VAS.

### 3.4. Side Effects of Ketamine

The systematic reviews featured in this umbrella review reported a set of adverse effects linked to ketamine use to alleviate acute pain during and after surgery, in emergency departments, and during procedures.

The side effects included in these reviews were dysphoria, delirium, hallucinations, headache, drowsiness, nightmares, nausea and vomiting, sedation, diplopia, urinary retention, respiratory depression, pruritus, deep vein thrombosis (DVT), and pulmonary embolism (PE).

The frequencies of several of these side effects in the perioperative settings underwent a meta-analysis, specifically nausea and vomiting, urinary retention, sedation, respiratory depression, hallucinations, and other psychotic events. The meta-analyses that compared incidence of these side effects of ketamine against control treated with placebo provided mixed results.

Studies by Bell et al. (2005 & 2006) [17,18] (*p* = 0.001), Brinck et al. (2018) [4] (from 27% to 24% (95% CI 16 to 54)), Hannon et al. (2023) [20] (RR 0.68; 95% CI, 0.50–0.92; I^2^ = 4.9%), Li and Chen (2019) [23], Xu et al. (2019) [32] (RR 0.67, 95% CI 0.51 to 0.89, *p* = 0.005), and Wang et al. (2020) [29] (OR = 0.54, 95% CI: 0.37 to 0.77, *p* = 0.0008) indicated that nausea and vomiting are less prevalent in patients treated with ketamine, compared with a placebo. However, the study by Pan et al. (2019) [25] found no significant difference in these outcomes (RR = 1.87, 95% CI = 0.65–3.10, *p* = 0.003). Regarding urinary retention, Hannon et al. (2023) [20] reported that the risk was not increased with ketamine use (RR 1.02; 95% CI, 0.53–1.94; I^2^ = 0.0%).

For psychotic events, the results varied. Brinck et al.’s (2018) [4] review found no significant difference in the incidence of delirium between the ketamine and control groups. Hannon et al. (2023) [20] reported no significant difference in the incidence of hallucinations (RR 0.70; 95% CI, 0.29–1.69; I^2^= 0.0%). However, Xu et al. (2019) [32] showed an increased incidence of psychotic events, which included drowsiness, nightmares, hallucinations, and delirium (RR 0.94; 95% CI 0.59 to 1.50; *p* = 0.79). Furthermore, ketamine was found to have lower sedation rates within 24 h after surgery compared with the control group (RR: 0.54, 95% CI 0.37 to 0.78, SDC 7).

The systematic reviews also reported various side effects of the ketamine therapy for managing acute pain in emergency care settings.

There was no significant differences in dizziness, nausea, and vomiting in the ketamine group compared with opioids, as reported by Balzer et al. (2021) [16] (10.7% vs. 11.1%, RR = 0.97, 95% CI: 0.63 to 1.49), Galili et al. (2023) [19] (RR 0.83, 95% CI 0.46 to 1.53), and Li (2021) [22] (dizziness, OR: 1.78; 95% CI: 0.54, 5.93; I^2^ = 43%; *p* = 0.34) (nausea and vomiting, OR: 1.47; 95% CI: 0.67, 3.20; I^2^ = 0%; *p* = 0.33). Additionally, studies indicated that there was no difference [16] (hypoxia: 3.9% vs. 14.4%, RR = 0.38, 95% CI = 0.10 to 1.41) and an insignificant decrease in the incidence of hypoxia and hypotension with ketamine use [19] (hypoxia: RR 0.23, 95% CI 0.04 to 1.35; hypotension: RR 0.21, 95% CI 0.02 to 1.81). However, ketamine was found to increase the risks of emergence reactions and dysphoria compared with the use of opioids alone, as noted by Galili et al. (2023) [19] (SDK at 0.15 mg/kg: RR 4.34, 95% CI 0.95 to 19.79; at 0.3 mg/kg: RR 12.01, 95% CI 2.32 to 62.06), Li (2021) [22] (OR: 5.67; 95% CI: 1.59, 20.24; I^2^ = 8%; *p* = 0.008).

Interestingly, when ketamine was used in combination with propofol, it was associated with lowered risks of respiratory events and overall adverse events compared with the use of propofol alone, as reported by Yan et al. (2015) [33] (from 35.4% to 29.0%, RR = 0.82, 95% CI = 0.68 to 0.99).

Xu et al. (2016) [31], while measuring the effectiveness of ketamine in managing CRPS, found that there were some side effects associated with the therapy. Specifically, patients that received ketamine exhibited mild-to-moderate psychosis-mimicking effects. Additionally, patients that received a 100 h infusion of ketamine had liver enzyme levels elevated to three times the upper normal limit, which increased the risks of liver injury.

The systematic review by Jalili et al. (2016) [21] compared the use of ketamine combined with propofol to propofol alone for managing acute pain in procedural sedation and analgesia. The findings demonstrated that ketamine significantly reduced the incidence of different negative effects. Specifically, the patients that received the combination of ketamine and propofol experienced lower rates of hypertension, nausea and vomiting, respiratory events, and emergence phenomena compared with those who received propofol alone.

## 4. Discussion

Acute pain is a substantial healthcare burden that is responsible for more than 70% of patients’ visits in the U.S. [35]. Given the strong need for efficient and safe acute pain management strategies, transition to multimodal pain management approaches, including nonstandard options, such as ketamine, is being actively considered. Ketamine was historically used primarily as an anesthetic agent during surgery and is now used for analgesia in the postoperative period in intensive care, postoperative care units, and emergency departments. The present umbrella review provided an overview on ketamine’s applications in acute pain therapy. The impact of ketamine on patients experiencing various forms of acute pain was investigated, covering 20 studies overall. The evidence supports the beneficial influence of ketamine on acute pain management.

Ketamine’s mechanism of action involves NMDA receptor antagonism and the modulation of pain pathways in the central nervous system. Ketamine primarily acts as an NMDA receptor antagonist [36]. This interaction decreases the activation of NMDA receptors, leading to a reduction in neuronal excitability, which plays a crucial role in its ability to alleviate pain. In addition, ketamine modulates pain pathways by interrupting the transmission of pain signals from the spinal cord to higher CNS centers. By affecting structures like the thalamus, medulla oblongata, and midbrain nuclei, ketamine further diminishes pain perception and enhances its overall analgesic efficacy [29]. Furthermore, ketamine enhances opioid-induced analgesia through its binding to spinal μ receptors, which increases the effectiveness of opioid signaling and supports an opioid-sparing effect [37].

The available literature does not provide enough evidence to determine the most effective route of administration or optimal dose of ketamine due to the broad variation in dosing and inconsistent efficacy outcomes. Our review identified studies that involved intravenous, intranasal, inhalation, intra-articular, and epidural forms of ketamine. While most research supported the efficacy of intravenous ketamine, the treatment durations varied from 30 min to 24 h, with doses that ranged from 0.01 mg/kg to 270 mg/70 kg.

Our findings parallel those of a recent meta-analysis of 15 RCTs, which suggested that ketamine was more effective in acute pain management in the early stages following treatment compared with morphine, specifically at 30 min after treatment, where the patients in the ketamine group had lower numeric rating scores than the patients in the morphine group. However, morphine was more efficient than ketamine 120 min after treatment, resulting in more prolonged pain reduction. In addition, in that meta-analysis, no statistically significant difference in the use of rescue analgesics was found between the ketamine and morphine groups [38]. The present review showed that ketamine can be associated with side effects, such as dysphoria, nausea, vomiting, and sedation. Our findings regarding the occurrence of side effects from ketamine compared with a placebo and other drugs are inconsistent, whereas Guo et al. (2024) [38] showed that the patients in the ketamine arm showed a lower frequency of complications necessitating treatment than those in the morphine group.

Due to the unique quality of ketamine, strong analgesic and sedative effects, and absence of significant cardiovascular and respiratory depression effects, it can be used for analgesia and sedation, not only in the operation room but also in the intensive care and postoperative care units, in the emergency department, in the ambulance during transportation, and even in battlefields. In contrast, other hypnotics, such as propofol, do not have strong analgesic properties, they also have a propensity to cause cardiovascular and respiratory depression. Therefore, ketamine can be used in such settings alone or in combinations with other hypnotics to improve the analgesic effects and patient’s stability [21].

Another benefit of ketamine is its versatility in administration, as it can be given intravenously, intramuscularly, orally, or even nasally. This range of administration routes expands the applicability of ketamine across different clinical settings and patient needs. For example, intranasal ketamine, when used in the emergency department for pain relief in patients with renal colic and acute trauma, was shown to provide adequate analgesia comparable with morphine [22]. The utility of ketamine, however, is constrained by its side effect profile. The side effects most commonly associated with higher doses of ketamine, including vomiting, nausea, hallucinations, vivid dreams, and dissociation, are a significant concern and often limit its use. In contrast, when low doses of ketamine are used for the treatment of acute pain, studies show that there is no significant difference in the incidence of side effects compared with morphine, suggesting that lower doses may offer an effective alternative with a similar safety profile [39].

Ketamine was also demonstrated its efficacy in total knee and total hip arthroplasties (TKA and TKA). It resulted in lower VAS scores within 48 h after surgery and lower opioid consumption within 48 h after surgery. There were no major side effects. Therefore, ketamine might be considered as an effective and safe analgesic for THA and TKA [29].

Several limitations of this umbrella review should be acknowledged. First, the interpretation of the results could be compromised due to the heterogeneity of the primary studies used for the present review. Differences in the dose, duration, and frequency of analgesic administration; patient population; diseases; and procedures could limit the accuracy of the results. Second, since the included reviews were published in English, important evidence on the effects of ketamine may have been missed in other languages. Third, as a result of AMSTAR 2, we found that the prevailing number of included reviews lacked proper methodological quality, which can introduce bias and lead to the misguided conclusions. Finally, ketamine has a relatively rapid onset of action, yet only five studies measured pain scores within 15 min of administration. In contrast, other studies recorded pain scores at varying intervals, ranging from hours to days, weeks, and even years. This discrepancy in timing posed a significant limitation in the primary studies, as it made it challenging to accurately capture the full extent of ketamine’s pain-relieving effects during the perioperative period.

The findings of this review have significant clinical implications. Ketamine’s capacity to lower opioid consumption may provide significant advantages for specific patient groups, such as those at risk for opioid dependence or opioid-induced side effects. This reduction has the potential to improve patient safety and minimize opioid-related complications. However, prolonged use of ketamine raises concerns about the risk of addiction and requires careful monitoring. Moreover, while ketamine demonstrates short-term efficacy, its practicality for long-term use is limited by the need for repeated dosing. In perioperative settings, infusion protocols for ketamine require close monitoring, especially when administered at anesthetic doses, as the risks associated with prolonged anesthesia are significantly heightened. Nevertheless, given the beneficial effect of ketamine in reducing acute pain and the need for opioid drugs, ketamine can serve as an alternative and appropriate adjunct for reducing opioid analgesic exposure. In addition, the use of ketamine can enhance the cost-effectiveness of treatment because it reduces the consumption of opioids.

Future research could focus on determining the optimal ketamine dose and treatment duration, as well as its long-term effects, to improve perioperative outcomes and pain management efficiency. Furthermore, given the substantial adverse effect of acute pain on the physical and mental health of patients, it is necessary to investigate the effects of ketamine therapy on the patients’ physical functioning and psychological impairments. The efficiency of ketamine use, along with non-pharmacological interventions, can be investigated. More evidence is required to demonstrate that ketamine may reduce postoperative hyperalgesia.

## 5. Conclusions

Intravenous ketamine administered in the perioperative setting was shown to reduce pain intensity and postoperative opioid consumption, with particularly notable effects in major orthopedic, abdominal, and thoracic surgeries. Additionally, ketamine delays the time to first request for analgesia in the non-stratified cohort. It was also demonstrated to reduce the incidence of postoperative nausea and vomiting. Moreover, when combined with other hypnotics, such as propofol, ketamine enhances the quality of analgesia and sedation, potentially mitigating the complications associated with propofol use alone. These findings suggest that intravenous ketamine could be a valuable adjunct in perioperative pain management, particularly for high-risk surgeries. 

Ketamine might also be used for acute pain management in the emergency department but more studies are required. Especially more RCTs focusing on the routes of administration are needed.

## Figures and Tables

**Table 1:** Basic characteristics of included systematic reviews.

PMID	Author (Year)	Research Type	Agents	Control	No. of Studies	No. of Patients	Acute Pain Type	Pain-Scoring Method	Ket Doses	Route of Administration	Reporting Guidelines
33098707	Balzer et al. (2021) [16]	SR with MA	LDK	Morphine	8	1191	ED	NPRS	0.2–0.5 mg/kg	IV	NA
16223384	Bell et al. (2005) [17]	SR with MA	Ketamine racemic, S(+), R(−)	Placebo, morphine	37	2137	Postop.	VAS and VRS	10 mg, 30 mg, 65 mg, 250–270 mg	IV bolus and/or infusion, EB, PCA, PCEA	QUOROM
16437490	Bell et al. (2006) [18]	SR with MA	Ketamine racemic, S(+), R(−)	Placebo, basic analgesics	37	2240	Postop.	VAS and VRS	10 mg, 30 mg, 65 mg, 250–270 mg	IV bolus and/or infusion, EB, PCA, PCEA	NA
30570761	Brinck et al. (2018) [4]	SR with MA	Ketamine racemic, S(+), R(−)	Placebo	130	8341	Before incision, during surgery, and postop.	VAS	0.25–1 mg (bolus), 2–5 μg/kg/min (infusion)	IV bolus, or infusion	MECIR
36972961	Galili et al. (2023) [19]	SR with MA	S(+) ketamine	Morphine	8	903	ED	NPRS	0.1–0.3 mg/kg (IV), 1.0–1.5 mg/kg (IN)	IV, IN, inhaled	PRISMA
36328104	Hannon et al. (2023) [20]	SR with MA	Ketamine	Placebo	7	NA	Intraop. and postop. THA and TKA	VAS, DN4	6 mcg/kg/min	IV infusion	PRISMA
26809929	Jalili et al. (2016) [21]	SR with MA	Ketamine-propofol	Propofol	18	2107	PSA	NA	NA	IV infusion	NA
33928616	Li et al. (2021) [22]	SR with MA	Ketamine	Placebo, opoid	7	1704	Pre-ED and ED	VAS	0.5–1 mg/kg	IN	NA
31446006	Li and Chen (2019) [23]	SR with MA	Ketamine	Placebo	6	244	Postop. TKA	VAS	3–6 μg/kg/min IV, 0.2–0.5 mg/kg IV, 0.25–2 mg/kg IA	IV infusion or bolus, IA	PRISMA
35065394	Meyer-Frießem et al. (2022) [24]	SR with MA	S(+) ketamine	Placebo, lower-dose ketamine	9	802	Intraop. and postop.	VAS	0.2–0.5 mg/kg IV, 1 mg/mL PCA, 100 mg PCIA	IV infusion or bolus, PCA, PCIA	PRISMA
31277113	Pan et al. (2019) [25]	SR with MA	Ketamine	Placebo	7	300	Postop. total knee arthroscopy	NPRS	0.5–1.0 mg/kg IA, 0.01–0.15 mg/kg IV	IA, IV infusion	PRISMA
28700455	Pendi et al. (2018) [26]	SR with MA	Ketamine	Placebo	14	649	Intraop. and postop. spine surgery	NPSR	0.15–1.00 mg/kg IV bolus 1.0–4.0 μg/kg/min	IV infusion and/or bolus, PCIA	NA
30842296	Thompson et al. (2019) [27]	SR with MA	NMDAR antagonists	Placebo, baseline control	70	1069	NA	NPRS	0.34 mg/kg	IV, IM, oral, subcutaneous	PRISMA
32214291	Wang et al. (2020) [28]	SR with MA	Ketamine	Placebo, other	20	1737	Cesarean section	VAS, NPRS	NA	IV, IS	NA
33080757	Wang et al. (2020) [29]	SR with MA	Ketamine	Placebo	21	1145	Postop. THA and TKA	VAS	0.1–0.6 mg/kg epidural, 0.05–0.5 mg/kg IV, 1.0–2.0 mg/kg PCIA	IV, IA, EB, PCIA	PRISMA
25234478	Wertli et al. (2014) [30]	SR with MA	IV or oral analgesics	Placebo, other	16	560	CRPS1	VAS, NPRS	7.2 μg/kg/min	IV	PRISMA
26891396	Xu et al. (2016) [31]	SR	IV analgesics	Placebo	63	NA	CRPS	VAS	1.2–7.2 μg/kg/min	IV	NA
31519671	Xu et al. (2019) [32]	SR with MA	S(+) ketamine	Placebo	10	577	Postop. THA and TKA	VAS	10 mg epidural, 0.25–2.0 mg/kg IA, 0.2–0.5 mg/kg IV bolus, 6 μg/kg/min IV infusion	IV infusion and/or bolus, IA, EB	PRISMA
26292077	Yan and McLeod (2015) [33]	SR with MA	Ketamine-propofol	Propofol	6	932	ED	NA	0.2–0.5 mg/kg	IV bolus	NA
29404715	Zhao et al. (2018) [34]	SR with MA	Ketamine racemic, S(+)	Baseline control	15	258	CRPS	NPRS, VAS	0.08–7.0 mg/kg/h	IV infusion	PRISMA

SR—systematic review, MA—meta-analysis, LDK—“low-dose ketamine”, ED—emergency department, NPRS—“numerical pain rating scale”, VAS—Visual Analog Scale, VRS—Verbal Rating Scale, intraop.—intraoperative, postop.—postoperative, NA—not available, IV—intravenous, IS—intraspinal, IA—intra-articular, IN—intranasal, EB—epidural bolus, PCA—“patient controlled analgesia”, PCEA—epidural patient-controlled analgesia, PCIA—“patient controlled intravenous analgesia”, PSA—“procedural sedation and analgesia”, THA—total hip arthroplasty, TKA—total knee arthroplasty, CRPS—“complex regional pain syndrome”, NMDAR—N-methyl-D-aspartate receptor.

**Table 2:** AMSTAR-2 quality ratings for included systematic reviews.

Author, Citation	1. Did the Research Questions and Inclusion Criteria for the Review Include the Components of PICO?	2. Did the Report of the Review Contain an Explicit Statement That the Review Methods Were Established Prior to the Conduct of the Review and Did the Report Justify Any Significant Deviations from the Protocol?	3. Did the Review Authors Explain Their Selection of the Study Designs for Inclusion in the Review?	4. Did the Review Authors Use a Comprehensive Literature Search Strategy?	5. Did the Review Authors Perform the Study Selection in Duplicate?	6. Did the Review Authors Perform the Data Extraction in Duplicate?	7. Did the Review Authors Provide a List of Excluded Studies and Justify the Exclusions?	8. Did the Review Authors Describe the Included Studies in Adequate Detail?	9. Did the Review Authors Use a Satisfactory Technique for Assessing the Risk of Bias (RoB) in Individual Studies That Were Included in the Review?	10. Did the Review Authors Report on the Sources of Funding for the Studies Included in the Review?	11. If a Meta-analysis Was Performed, Did the Review Authors Use Appropriate Methods for the Statistical Combination of Results?	12. If a Meta-analysis Was Performed, Did the Review Authors Assess the Potential Impact of the RoB in Individual Studies on the Results of the Meta-analysis or Other Evidence Synthesis?	13. Did the Review Authors Account for the RoB in Individual Studies When Interpreting/ Discussing the Results of the Review?	14. Did the Review Authors Provide a Satisfactory Explanation for, and Discussion of, Any Heterogeneity Observed in the Results of the Review?	15. If They Performed Quantitative Synthesis, Did the Review Authors Carry Out Adequate Investigation of the Publication Bias (Small Study Bias) and Discuss Its Likely Impact on the Results of the Review?	16. Did the Review Authors Report Any Potential Sources of Conflict of Interest, Including Any Funding They Received for Conducting the Review?
Galili et al. (2023) [19]	+	+	−	+	+	+	−	+	+	−	+	−	−	+	+	+
Hannon et al. (2023) [20]	+	−	+	Partially yes	+	NG	−	−	Partial yes	−	+	−	−	−	−	+
Meyer-Frießem et al. (2022) [24]	+	+	−	Partially yes	+	+	−	−	+	−	+	+	+	+	+	+
Li et al. (2021) [22]	+	−	−	Partially yes	+	NG	−	Partial yes	+	−	+	+	+	+	+	−
Balzer et al. (2021) [16]	+	−	−	Partially yes	+	+	−	Partial yes	+	−	+	+	+	+	+	−
Wang et al. (2020) [28]	+	−	−	Partially yes	+	+	−	Partial yes	Partial yes	−	+	−	−	−	−	−
Wang et al. (2020) [29]	+	−	−	Partially yes	+	+	−	Partial yes	Partial yes	−	+	−	−	+	+	+
Xu et al. (2019) [32]	+	−	−	Partially yes	+	+	−	+	Partial yes	−	+	−	−	+	+	+
Li and Chen (2019) [23]	+	−	−	Partially yes	NG	+	−	+	+	−	+	+	+	+	−	+
Pan et al. (2019) [25]	+	−	−	Partially yes	+	+	−	+	Partial yes	−	+	−	−	+	−	−
Thompson et al. (2019) [27]	+	−	−	Partially yes	+	+	−	Partial yes	Partial yes	−	+	−	−	+	+	+
Brinck et al. (2018) [4]	+	+	−	Partially yes	+	+	+	Partial yes	+	+	+	+	+	+	+	+
Zhao et al. (2018) [34]	+	+	−	Partially yes	+	+	−	+	−	−	+	−	−	−	+	+
Pendi et al. (2018) [26]	+	−	−	Partially yes	+	−	−	−	−	−	+	−	−	−	−	−
Xu et al. (2016) [31]	+	−	−	Partially yes	+	+	−	−	−	−	N/A	N/A	−	−	N/A	+
Jalili et al. (2016) [21]	+	−	−	Partially yes	+	NG	−	Partial yes	Partial yes	−	+	−	−	−	+	+
Yan and McLeod (2015) [33]	+	−	−	Partially yes	NG	NG	−	Partial yes	+	−	+	+	+	+	+	−
Wertli et al. (2014) [30]	+	−	−	+	+	NG	−	+	Partial yes	−	+	−	−	−	−	−
Bell et al. (2005) [17]	+	+	−	Partially yes	+	NG	+	Partial yes	−	−	+	−	−	+	−	+

“+” indicates that the criterion is met adequately. “−”indicates that the criterion is not met or is inadequately addressed.

## Data Availability

Not applicable.

## Abbreviations

CNS	Central nervous system
CRPS	Complex regional pain syndrome
DN4	Douleur Neuropathique 4
DVT	Deep vein thrombosis
EB	Epidural bolus
ED	Emergency department
IA	Intra-articular
IN	Intranasal
Intraop	Intraoperative
IS	Intraspinal
IV	Intravenous
LDK	Low-dose ketamine
MA	Meta-analysis
MECIR	Methodological Expectations of Cochrane Intervention Reviews
NA	Not available
NMDA	N-methyl-D-aspartate
NMDAR	N-methyl-D-aspartate receptor
NPRS	Numeric Pain Rating Scale
NSAIDs	Nonsteroidal anti-inflammatory drugs
PCA	Patient-controlled analgesia
PCEA	Epidural patient-controlled analgesia
PCIA	Patient controlled intravenous analgesia
PE	Pulmonary embolism
Postop	Postoperative
PPSP	Persistent postsurgical pain
PSA	Procedural sedation and analgesia
SDK	Single-dose ketamine
SR	Systematic review
THA	Total hip arthroplasty
TKA	Total knee arthroplasty
VAS	Visual Analog Scale
VRS	Verbal Rating Scale
